# Supplementary material for: Overcoming confounding plate effects in differential expression analyses of single-cell RNA-seq data
Source: Biostatistics. 2017 Feb 6;18(3):451–64. doi: 10.1093/biostatistics/kxw055 (PMC5862359; doi:10.1093/biostatistics/kxw055)
Supplement: Supplementary Data [file kxw055_supp.pdf]

Overcoming confounding plate effects in differential expression analyses of single-cell RNA-seq data

Supplementary Materials

by

Aaron T. L. Lun[1] and John C. Marioni[1,2,3]

[1]Cancer Research UK Cambridge Institute, University of Cambridge, Li Ka Shing Centre, Robinson Way, Cambridge CB2 0RE, United Kingdom

[2]EMBL European Bioinformatics Institute, Wellcome Genome Campus, Hinxton, Cambridge CB10 1SD, United Kingdom

[3]Wellcome Trust Sanger Institute, Wellcome Genome Campus, Hinxton, Cambridge CB10 1SA, United Kingdom

December 10, 2016

1 Details on the simulation design

1.1 Overview

We simulate from a NB-log-normal mixture, where the counts are conditionally NB-distributed and the plate effect is log-normally distributed. Define Y_{ijkg} as the random variable for the count of gene i in cell j in plate k of group g, θ_{jkg} as the random variable for the bias in cell j in plate k of group g, and δ_{ikg} as the random variable for the gene/plate effect in gene i and plate k of group g. Y_{ijkg} is conditionally distributed as

$$Y_{ijkg}|\delta_{ikg}, \theta_{jkg} \sim \text{NB}(\delta_{ikg}\theta_{jkg}\mu_{ig}, \varphi_i)$$

where μ_{ig} is the expected read count of gene i in group g and φ_i is the NB dispersion for gene i. All counts are assumed to be independently sampled, conditional on the realized values of θ_{jkg} and δ_{ikg}.

We assume that $\log(\delta_{ikg})$ is normally distributed with variance σ^2 and mean $-\sigma^2/2$, such that $E(\delta_{ikg}) = 1$, i.e., the plate effect for each gene averages out across many plates. Similarly, θ_{jkg} is sampled from some (empirically defined, see below) distribution with a mean of unity, i.e., the bias averages out across many cells. We also assume that θ_{jkg} and δ_{ikg} are independent of each other. Having means of unity for both θ_{jkg} and δ_{ikg} is not strictly necessary but simply ensures that $E(Y_{ijkg}) = \mu_{ig}$, consistent with its definition.

1.2 Parameter estimation from real data

We designed our simulation to mimic the characteristics of the mESC data set (Kolodziejczyk *and others*, 2015). This required some pre-processing to remove irrelevant genes and cells from the data prior to parameter estimation. Specifically, low-abundance genes with average counts below 1 were filtered out. Also, cells were only used if they belonged to a batch that contained all three culture types, i.e., serum, a2i and 2i.

We then estimated the simulation parameters from the data. For the cell-specific biases, we applied the deconvolution method (Lun *and others*, 2016) where all cells corresponding to each culture type were defined as a cluster. This yielded size factors centred around unity (Figure S1a), which can be treated as estimates of θ_{jgk} for all cells. To estimate the mean count of each gene, we fitted an intercept-only GLM to the counts from all cells, using the mglmOneGroup function in edgeR with mean-centred log-size factors as offsets. The resulting coefficient was used to define μ_{i0}, i.e., the expected count of gene i (Figure S1b). For non-DE genes, μ_{ig} was set to μ_{i0} for all groups, whereas for DE genes, $\mu_{ig} = \psi_{ig}\mu_{i0}$ for some fold change ψ_{ig}. Finally, we used the estimateDisp function in edgeR to estimate the NB dispersion. As the NB distribution is conditional on the plate-specific mean, we treated each plate as a separate group during GLM fitting and dispersion estimation. The "tagwise" dispersion estimate was used as φ_i for each gene (Figure S1c).

To estimate the variability of the plate effect, we fitted a GLMM to the counts for each gene using the glmer function in the lme4 package (Bates *and others*, 2015). We set the culture type and batch as fixed effects, the plate of origin as a random effect, and log-size factor as the offset for each cell. The distribution family was set to a NB distribution with a size (i.e., "theta") parameter of φ_i^{-1} for gene i. After fitting, the estimated variance of the random effect was obtained with the VarCorr function (Figure S1d). As the NB GLMM uses a log link function, this estimate represents the variance of the log-transformed plate effect and thus can be used as σ^2 in our simulation. However, each gene-specific estimate will be unstable with small numbers of plates. We improved precision by using the average estimate across all genes as σ^2. Note that the large number of zeroes in Figure S1d is likely caused by applying non-negativity constraints to unstable estimates, and does not indicate that the true variance of the plate effect is zero for those genes.

As an alternative to the NB model, we fitted a zero-inflated NB (ZINB) distribution to the counts for each gene. This was done using the zeroinfl function from the pscl package (Zeileis *and others*, 2008). The plate of origin was used as a factor for the NB component, whereas a common zero component was used for all cells to simplify regression. Offsets were defined as log-transformed size factors. For the NB component, the mean count was computed for each plate and the grand mean μ'_{i0} across plates was computed for each gene. The NB dispersion φ'_i and the probability p'_i of belonging to the zero component were also estimated for each gene. These values were used to redefine the sampling distribution of Y_{ij} as a ZINB distribution. Specifically, the mean of the NB component was set to $\delta_{ikg}\theta_{jgk}\mu'_{i0}$, the dispersion was set to φ'_i and the zero probability was set to p'_i. (For simplicity, we do not consider DE when simulating ZINB counts.)

1.3 Defining simulation scenarios

We simulated data for a scRNA-seq experiment with two groups of three plates. The number of cells on each plate was sampled from a discrete uniform distribution on $[50, 100]$. The bias θ_{jgk} for each cell was sampled with replacement from the set of size factor estimates obtained from the mESC data set. We then randomly selected 10000 genes from the mESC data. (For assessing type I error control, all genes are non-DE such that the null hypothesis is true for each gene.) For each chosen gene, the plate effect δ_{ikg} in plate k of group g was sampled from a log-normal distribution with parameters defined from the mESC data. This, along with the mean and dispersion of the chosen gene, was used to define the sampling distribution of Y_{ijkg} for each cell on the plate. We repeated this process for each plate to obtain counts for all genes in all cells.

We tested different scenarios by repeating the simulation with modified parameters. To reduce the variability of the plate effect, we halved the estimate of σ^2 prior to sampling δ_{ikg}. Similarly, we set $\sigma^2 = 0$ to generate data where the plate effect was completely absent. We increased the variability in the number of cells per plate, by sampling the number per plate from a Uniform(20, 100) distribution. We also increased the variability in the size factors, by multiplying the size factor for cell j by 2^{V_j} where $\log_2(V_j)$ is a normally distributed random variable with variance 0.25. This effectively increases the variance of the log-size factors across cells by 0.25, from the current variance of around 0.5 in Figure S1a. Next, we replaced the NB distribution with a ZINB distribution (with parameters estimated from real data, as previously described) for count sampling. Finally, we increased the size of the data set by using six plates per group.

To evaluate power and FDR control, we incorporated DE genes into the simulation. We chose a random set of G genes, which was partitioned into two further subsets of equal size. One subset of genes was upregulated in the first group of plates, whereas the other subset was upregulated in the second group. Recall that, for a DE gene i, $\mu_{ig} = \psi_{ig}\mu_{i0}$ for some group-/gene-specific fold change ψ_{ig}. Let the constant ψ_0 represent the overall DE fold change between groups. For all genes in the first subset, we define $\psi_{i1} = \sqrt{\psi_0}$ and $\psi_{i2} = 1/\sqrt{\psi_0}$ to introduce upregulation in the first group. Similarly, for genes in the second subset, we define $\psi_{i2} = \sqrt{\psi_0} = \psi_{i1}^{-1}$ to introduce upregulation in the second group. We performed these simulations with $\psi_0 = 3$ and $G = 2000$ by default. However, we also tested scenarios with σ^2 set to 0, to eliminate the plate effect; ψ_0 set to 6, to obtain stronger DE fold changes; or G set to 4000, to obtain more DE genes.

In all scenarios, we added balanced DE (i.e., an equal amount of up-/downregulation per group) to avoid introducing composition bias (Robinson and Oshlack, 2010). This simplifies the downstream analyses since library size normalization can be used with all methods. (The library size for a cell is defined as the sum of its counts across all genes. Library size normalization involves dividing counts by the library size, to correct for differences in sequencing depth between cells.) Thus, any difference in performance between analyses cannot be attributed to a difference in normalization accuracy for single-cell or summed counts. This is an important subtlety, as bulk normalization methods fare poorly on scRNA-seq data with many zero counts (Lun *and others*, 2016). If these methods are used, they may contribute to the loss of error control.

2 Detailed description of the DE analysis

2.1 Implementation of the analysis methods

For edgeR v3.16.4, DE analyses were performed with and without EB shrinkage. In the first analysis, an abundance-dependent trend was fitted to the NB dispersions, and the trended NB dispersion was used to fit a GLM for each gene (McCarthy *and others*, 2012). The GLM deviance was used to estimate the QL dispersion for each gene, which was stabilized by robust EB shrinkage towards a mean-QL dispersion trend (Lund *and others*, 2012; Phipson *and others*, 2016). DE testing between groups was performed using the QL F-test. In the second analysis, the NB dispersion was estimated for each gene individually. This was used directly to fit the GLM for each gene, and DE testing for each gene was performed using the LRT.

For voom, counts were converted to log-CPM values and precision weights were computed from a fitted mean-variance trend. A linear model was fitted to the log-CPMs and weights for each gene using methods in the limma package v3.30.6. Sample variances were stabilized by robust EB shrinkage, and genes were tested for DE between groups using a moderated t-test. This analysis was also repeated after using the duplicateCorrelation function (Smyth *and others*, 2005) to estimate the correlations introduced by the plate effects. For each gene, this correlation represents the strength of the association between the count for each

cell and its plate of origin. The consensus correlation across all genes was estimated and used for generalized least squares fitting with a plate blocking factor. DE testing was then performed on this refitted model.

For DESeq2 v1.14.0, the NB dispersion was estimated for each gene and a mean-dispersion trend was fitted across genes. A normal prior was applied to the log-dispersions for all genes, and the maximum *a posteriori* estimate of the NB disperison was obtained for each gene. The estimated dispersion was used to fit a GLM to the counts for each gene. DE testing was performed between groups using the Wald test.

For Monocle v2.2.0, counts were transformed into CPM values that were considered to be roughly log-normal. These were used for DE testing between groups with the LRT in the differentialGeneTest function.

For MAST v1.0.5, counts were converted into CPMs and $\log_2$-transformed after adding a pseudo-count of 1. For each gene, a hurdle model was fitted to the log-CPMs across all cells using the zlm.SingleCellAssay function with default parameters. In this model, the group was set as the factor of interest and the proportion of genes with non-zero counts in each library was used as an additional covariate (Finak *and others*, 2015). Putative DE genes between groups were identified using the LRT implemented in the lrTest function.

The glmer.nb function in the lme4 package v1.1-12 was used to fit a NB GLMM to the counts for each gene. The group was set as a fixed effect while the plate of origin was set as a random effect. Offsets were defined as the log-transformed library sizes. To detect DE between groups, the GLMM was refitted using a null design without the group factor. A LRT was then performed using the full and null model fits.

2.2 Explanation of the normalization strategy

For any given set of counts, the same normalization procedure was used for all analyses. For simulated data, size factors were defined as the mean-centred library sizes, calculated by dividing all library sizes by the mean library size across all cells. This represents library size normalization, which is appropriate due to the presence of balanced DE as previously described. The mean centering ensures that the size factors are centred around unity, which is required for DESeq2 to yield sensible normalized counts. (Scaling all of the size factors by a constant is permissable as it does not affect the relative normalization between cells.) For real single-cell data, size factors were computed with the deconvolution method (Lun *and others*, 2016). To mitigate any DE, cells were clustered by culture type in the mESC data set prior to normalization. For real summed data, size factors were defined using the DESeq normalization method (Anders and Huber, 2010). In each scenario, size factors were either used directly (e.g., in the DESeq2 analysis) or converted into effective library sizes to compute offsets or CPM values. The conversion was performed by scaling each size factor by the mean of the original library sizes across all cells/plates, to recover the scale of the original sizes.

Normalization on the count sums is necessary as it eliminates plate-specific biases. These biases are driven by different numbers of cells per plate, different library sizes of those cells, as well as composition biases caused by DE between cells. In fact, the plate-specific bias is equal to the sum of the cell-specific biases across all cells on the plate. (This reasoning is based on the pooling normalization framework in Lun *and others* (2016), after setting $t_j = 1$ for each cell j.) The bias will systematically alter the mean count for all genes on the plate, requiring normalization to avoid detection of spurious differences between plates.

2.3 Using plates as fixed effects is not appropriate

One strategy to account for plate effects in the DE analysis is to treat the plate factor as a fixed effect. This means that each plate has an explicit term representing their average expression in the linear model, which absorbs any plate-specific effect for each gene. DE analyses between plates in different groups could then be performed by computing the average of the terms within each group, and comparing those averages between groups. However, the variance/dispersion estimate will be conditional on the fitted values of the plate-specific terms. This means that the plate-to-plate variability in the plate effect will not be modelled. Thus, the inferences from this analysis cannot be generalized to a repeated experiment with a new set of plates (and a different set of plate effects). This is unsatisfactory as the DE results will not be replicable.

3 Summation and plate-specific mean variance relationships

Data sets will contain different numbers of cells in each plate, as well as different library sizes across cells. This means that the count sum for each plate will not be identically distributed. The most obvious difference

is observed in the magnitude of the count sum between plates with different numbers of cells or library sizes per cell. This leads to a consistent fold-difference in the expression of all genes on one plate relative to another, which can be resolved by normalizing on the total library size for each plate (i.e., the sum of count sums across genes) or with related methods. The greater problem is that the count sum for each plate will not have the same mean-variance relationship. Briefly, the variance of common count distributions can be expressed as a function of the mean. Most parametric models assume that this function is the same for all observations of a given gene. For example, edgeR and DESeq2 use a single NB dispersion estimate for each gene, which defines an identical mean-variance relationship for all counts of that gene. However, if the count sum for each plate has a different relationship, the accuracy of the models may be compromised.

In practice, differences in library sizes and/or numbers of cells across plates do not seem to negatively affect performance on summed counts. Power and error control are maintained in our simulations with variable library sizes and cell numbers. The robustness of summation can be explained by examining the mean-variance relationship of the simulated data. For simplicity, partition the cells on plate k of group g into B_{kg} bins. (This does not affect generality, as any number of arbitrary bins can be defined.) Each bin b contains N_{bkg} cells, all of which have a constant bin-specific bias t_{bkg} that scales the mean count for all genes. Specifically, let $\lambda_{ibkg} = \delta_{ikg} t_{bkg} \mu_{ig}$ for gene i in each cell of bin b in plate k in group g. The count for each cell is independently sampled from a NB distribution with mean λ_{ibkg} and dispersion φ_i, as described in the simulation design. The conditional variance of the count sum S_{ikg} for plate k in group g is

$$\text{var}(S_{ikg}|\delta_{ikg}) = \sum_{b=1}^{B_{kg}} N_{bkg}\lambda_{ibkg} + \varphi_i \sum_{b=1}^{B_{kg}} N_{bkg}\lambda_{ibkg}^2 \ .$$

In the following text, all summations with an index of b are calculated across all B_{kg} bins in plate k of group g. As $E(S_{ikg}) = \sum_b N_{bkg}\lambda_{ibkg} = \sum_b N_{bkg} t_{bkg} \mu_{ig}$, the variance of the count sum can be decomposed to

$$\begin{aligned}
\text{var}(S_{ikg}) &= \text{var}\{E(S_{ikg}|\delta_{ikg})\} + E\{\text{var}(S_{ikg}|\delta_{ikg})\} \\
&= \text{var}\left(\delta_{ikg}\sum_b N_{bkg} t_{bkg}\mu_{ig}\right) + E\left(\delta_{ikg}\sum_b N_{bkg} t_{bkg}\mu_{ig} + \varphi_i \delta_{ikg}^2 \sum_b N_{bkg} t_{bkg}^2 \mu_{ig}^2\right) \\
&= \left(\sum_b N_{bkg} t_{bkg}\mu_{ig}\right)^2 \text{var}(\delta_{ikg}) + \left(\sum_b N_{bkg} t_{bkg}\mu_{ig}\right) + \left(\varphi_i \mu_{ig}^2 \sum_b N_{bkg} t_{bkg}^2\right) E(\delta_{ikg}^2) \\
&= E(S_{ikg})^2 \text{var}(\delta_{ikg}) + E(S_{ikg}) + \left\{\varphi_i \frac{\sum_b N_{bkg} t_{bkg}^2}{(\sum_b N_{bkg} t_{bkg})^2}\right\} E(S_{ikg})^2 E(\delta_{ikg}^2) \ .
\end{aligned}$$

Note that $E(\delta_{ikg}^2)$ and $\text{var}(\delta_{ikg})$ are constant for all plates. This means that, in the above expression, only the third term defines the plate-specific aspect of the mean-variance relationship. Now, consider the behaviour of this term as the number of cells increases. If N_{bkg} increases in each of n bins, the limit becomes

$$\lim_{\substack{N_{b_1 kg}\to\infty \\ \cdots \\ N_{b_n kg}\to\infty}} \frac{\sum_b N_{bkg} t_{bkg}^2}{(\sum_b N_{bkg} t_{bkg})^2} = \sum_b \lim_{\substack{N_{b_1 kg}\to\infty \\ \cdots \\ N_{b_n kg}\to\infty}} \frac{N_{bkg} t_{bkg}^2}{(\sum_b N_{bkg} t_{bkg})^2} = 0$$

for positive t_{bkg}. For any non-zero value of $\text{var}(\delta_{ikg})$, the relative contribution of the plate-specific term will approach zero as the number of cells increases. Thus, the mean-variance relationship of the count sum will be similar between plates with many cells, regardless of the number of cells or their library sizes.

4 Benefits of hiding the variability between cells

If more cells contribute to the sum, variability between cells will be hidden as it will no longer affect the total variance. However, this is not undesirable for single-cell analyses. Genes with reproducible DE between plates should not be penalized for having high variability within plates, e.g., due to cellular heterogeneity or the presence of subpopulations. This philosophy is almost the exact opposite of that in microarray analyses

involving technical replicates, where variability between replicates is explicitly modelled rather than being hidden by averaging the replicate signals (Smyth *and others*, 2005). The difference in strategies is due to the fact that the technical replicates in microarray analyses are expected to be similar. Genes with large differences between replicates are considered to be unreliable and should have reduced significance for DE. In contrast, cells are not expected to be similar due to biological heterogeneity within populations. Large cell-to-cell variability has no bearing on the reliability of DE between populations when many cells are present. For example, if the subpopulation structure is the same in each replicate plate, one would have a situation with large variability between cells in different subpopulations but reproducible count sums across plates.

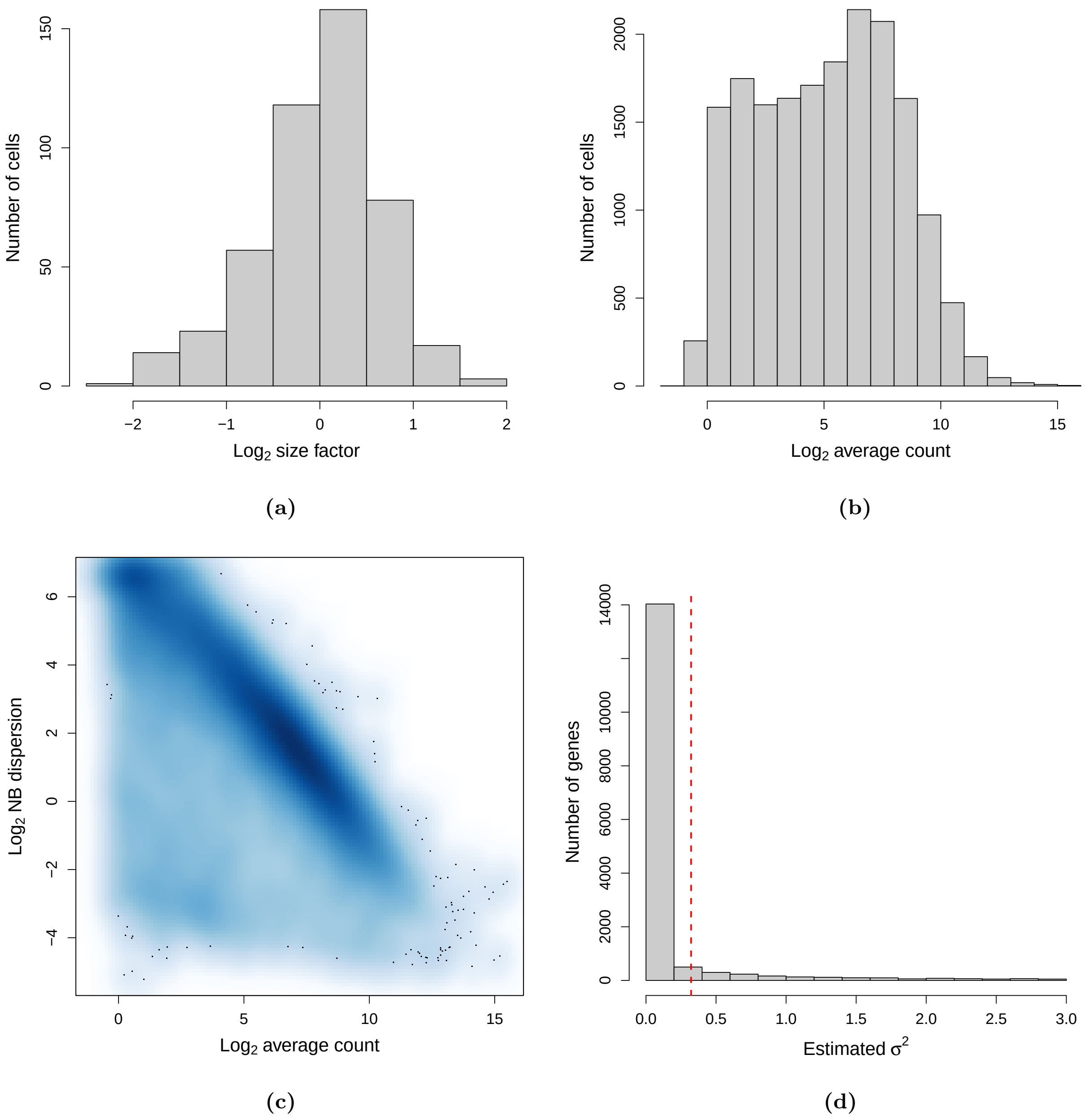

Figure S1: Estimates of the simulation parameters from the mESC data set. (a) Histogram of size factors across all cells. (b) Histogram of log-mean counts across all genes. (c) Gene-specific dispersion estimates, plotted against the log-means for all genes. (d) Histogram of the estimated variances of the plate effect across all genes, where the red line indicates the average (estimated at 0.32 in this data set).

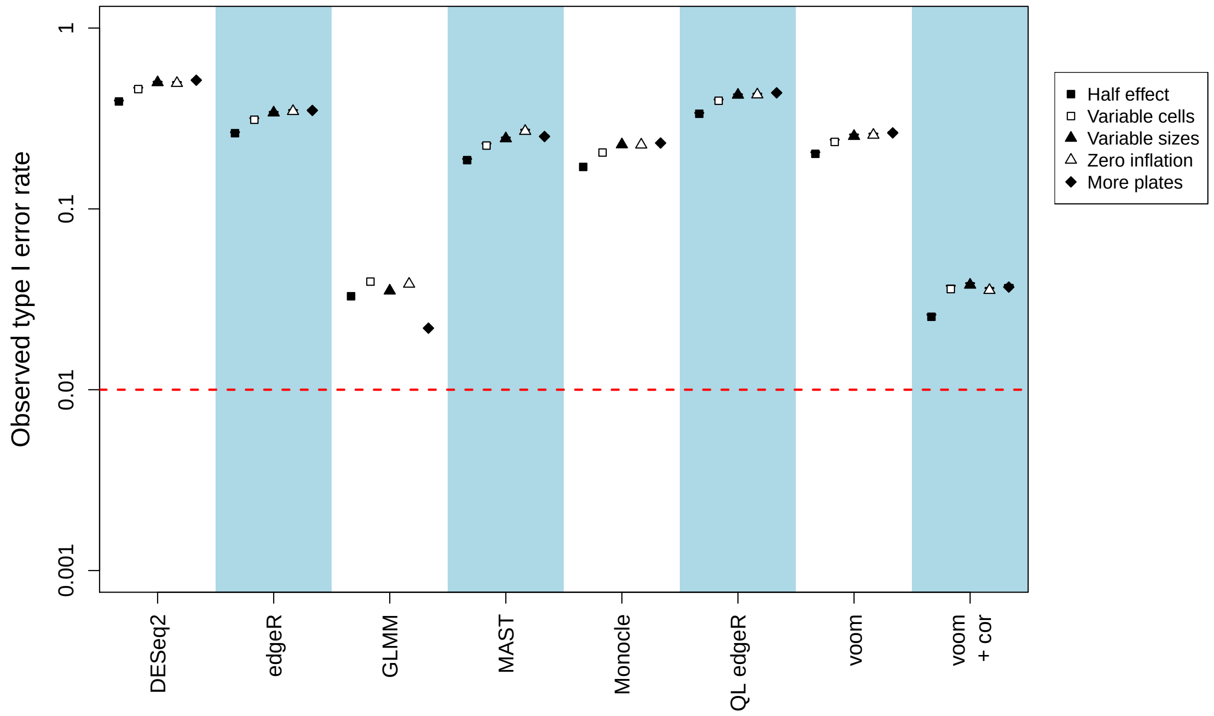

Figure S2: Observed type I error rates for each method on simulated data upon halving the variability of the plate effect; increasing the variability in the number of cells per plate; increasing the variability in library sizes across cells; using a ZINB distribution to sample counts; and increasing the number of plates in each group. Error rates are shown on a log scale and represent the average across 10 simulation iterations. Each error bar represents the standard error of the log-average rate. The threshold of 0.01 is represented by the red line. Only one iteration was used for Monocle and GLMMs due to their long run times.

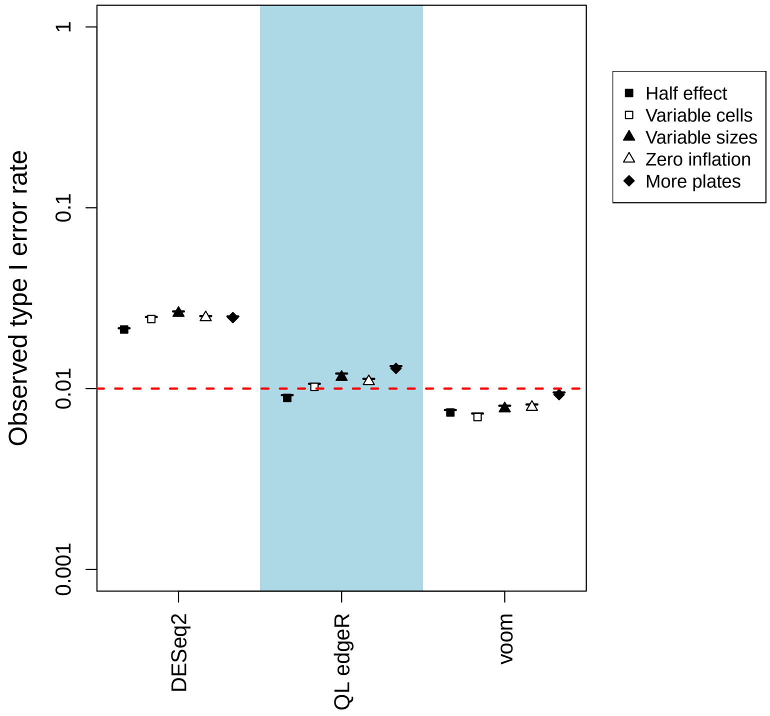

Figure S3: Observed type I error rate for each method after summation in a variety of simulation scenarios (see Figure S2). Error rates are shown on a log scale and represent the average across 10 simulation iterations. Error bars represent standard errors, and the threshold of 0.01 is represented by the red dashed line.

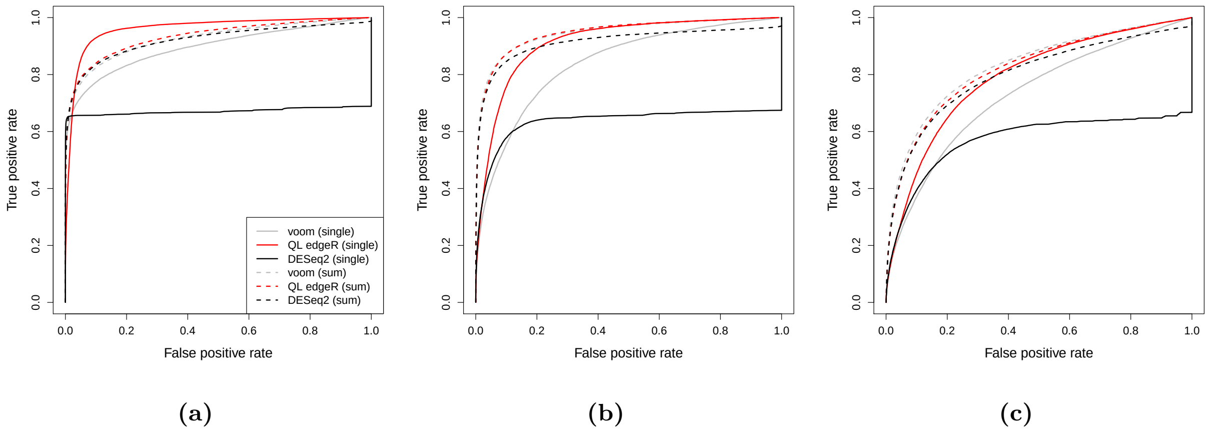

Figure S4: ROC curves for each analysis method using single-cell (full) and summed counts (dashed), in simulations with (a) no plate effect, (b) larger DE fold changes and (c) more DE genes. Curves are shown for DESeq2 (black), voom (grey) and QL edgeR (red). Each curve represents the average of 10 iterations.

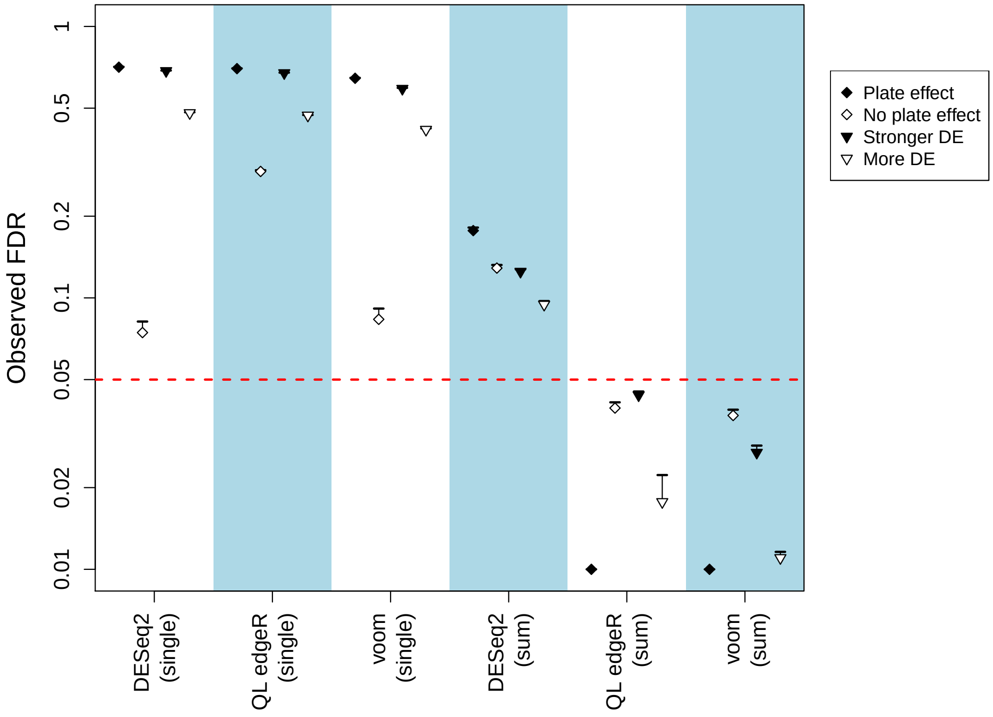

Figure S5: Observed FDRs for each analysis method using single-cell or summed counts in simulations with DE genes and a plate effect (denoted above as "Plate effect"). Settings for additional simulation scenarios are equivalent to those used in Figure S4. The red line represents the nominal threshold of 5%. All values represent the average of 10 simulation iterations, and error bars represent the standard error.

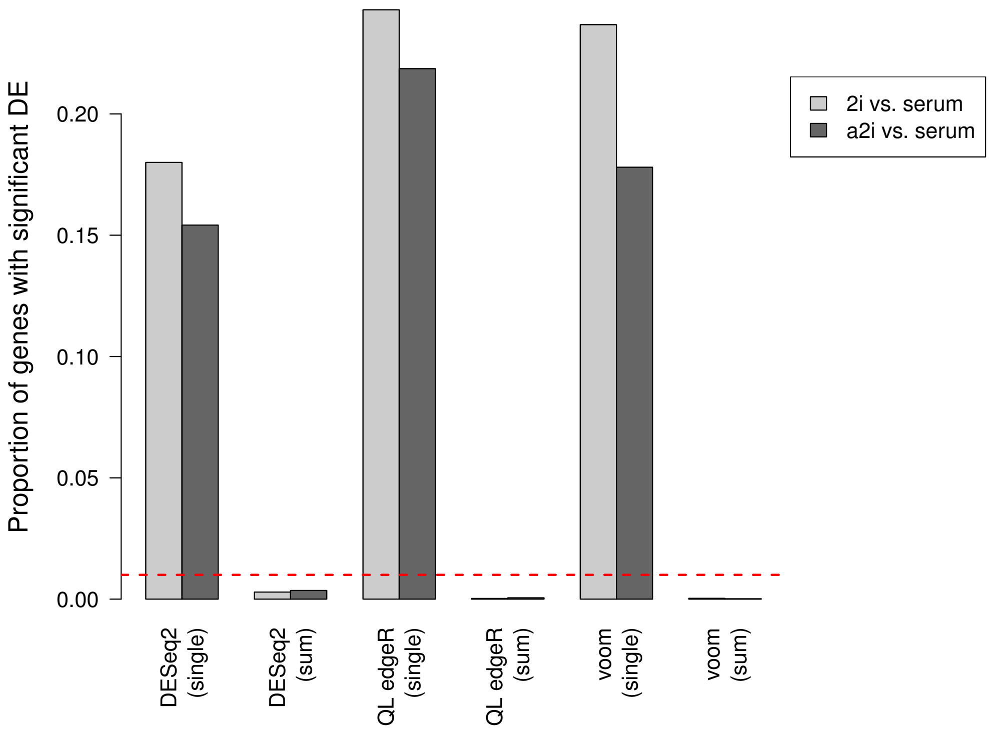

Figure S6: Proportion of genes with p-values below the nominal threshold of 0.01 (red) for the 2i vs. serum and a2i vs. serum comparisons, upon applying each method with and without summation to label-swapped data. Each proportion represents a type I error rate as the null hypothesis is true for each gene.

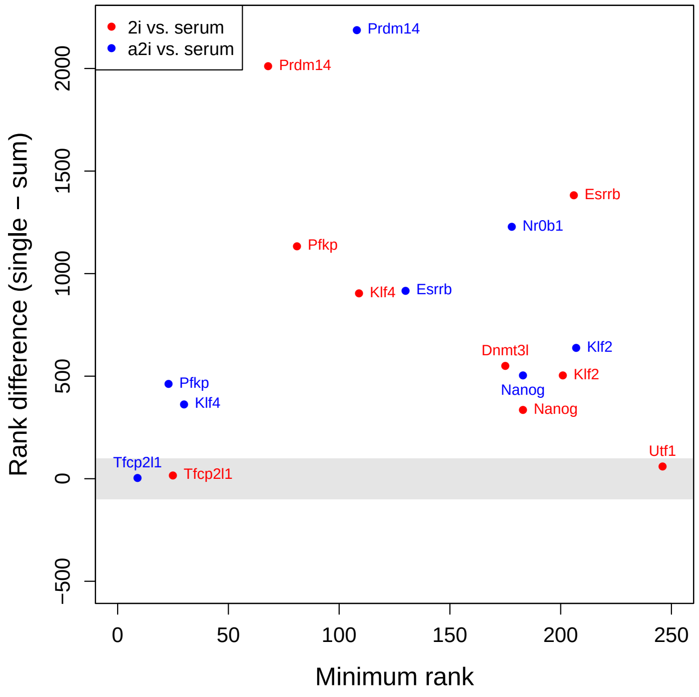

Figure S7: Difference in ranks between DE analyses on single-cell and summed counts, for key pluripotency factors listed in Figure S5 of Kolodziejczyk *and others* (2015). For each comparison between culture types, the rank for each gene was obtained from the DE lists of QL edgeR using either summed or single-cell counts. A positive difference in ranks means that the gene is lower ranked (i.e., further down the list) in the single-cell analysis compared to the summed analysis. The minimum rank is the smaller rank from either analysis, and represents the "best" position of each gene. The grey bar marks a difference in ranks of ± 100. The difference in the enrichment of factors in the top 250 genes between summed and single-cell analyses was tested using Fisher's exact test, yielding a p-value of 1.9×10^{-4} (using total numbers of genes across both comparisons).

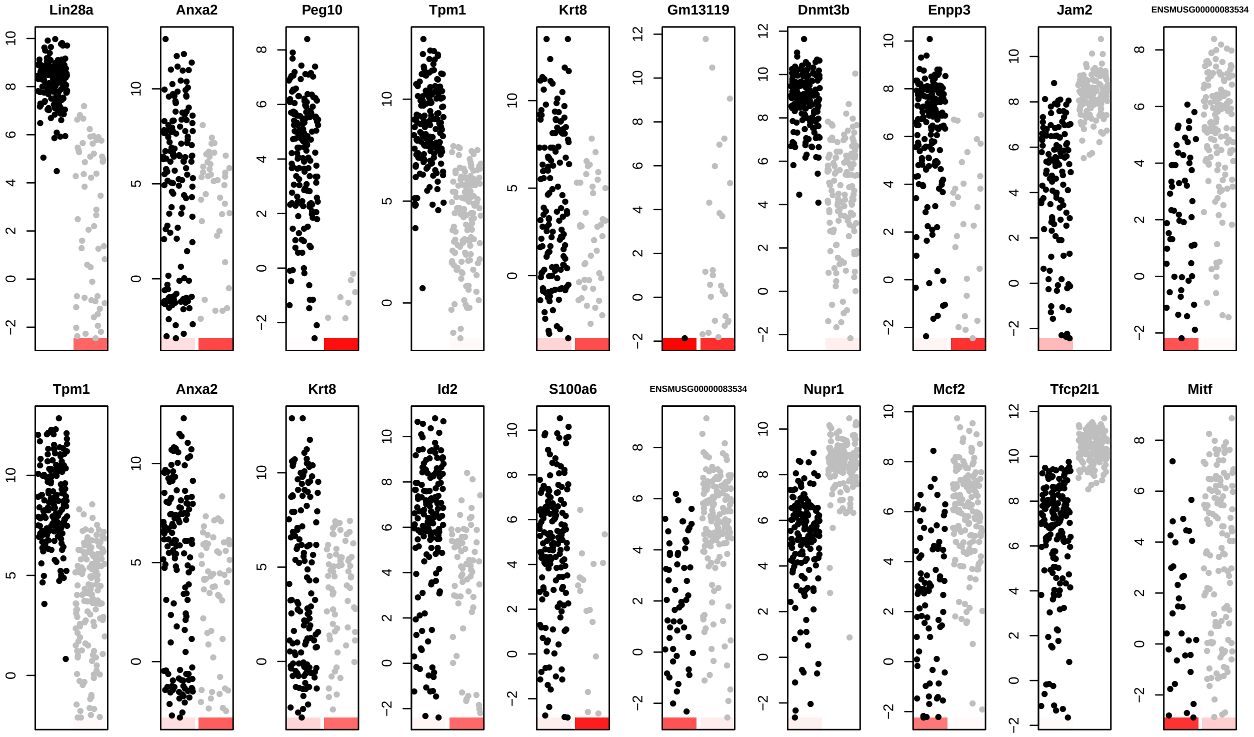

Figure S8: Expression profiles of DE genes between 2i and serum (top) or between a2i and serum (bottom) in the mESC data set, for the top genes detected by QL edgeR on summed counts. Gene expression is quantified in terms of the log-CPM for each cell in the serum (black) or a2i/2i groups (grey), after removing the batch effect with the removeBatchEffect function in limma. Log-CPMs for zero counts are not shown – rather, the intensity of colour in the red bars is equal to the proportion of zeroes in each group.
